# Supplementary material for: Analysis of single-cell RNA sequencing data based on autoencoders
Source: BMC Bioinformatics. 2021 Jun 8;22:309. doi: 10.1186/s12859-021-04150-3 (PMC8186186; doi:10.1186/s12859-021-04150-3)
Supplement: Supplementary file 1 — Additional file 1. Mathematical formulation of the proposed autoencoders. We provide the mathematical derivation of GMMMD and GMMMDVAE as well as the generalised formulation that we derived by following the notation proposed in [35]. [file 12859_2021_4150_MOESM1_ESM.pdf]

Analysis of single-cell RNA sequencing data based on autoencoders

Andrea Tangherloni, Federico Ricciuti, Daniela Besozzi, Pietro Liò, Ana Cvejic

Mathematical formulation of the proposed autoencoders

In what follows, we use the notation proposed in [1] to derive the extension of the Mean Maximum Discrepancy Autoencoder (MMDAE) and Mean Maximum Discrepancy Variational Autoencoder (MMDVAE) [1] with multiple Gaussian distributions.

$p^*(\mathbf{x})$ is the unknown probability in the input space over which the optimisation problem is formulated. A similarity measure $\mathcal{L}$ between the input and the output of the autoencoders (AEs) with respect to the distribution $p^*(\mathbf{x})$ is maximised:

$$\arg\max_{\boldsymbol{\phi},\boldsymbol{\theta}} \mathbb{E}[\mathcal{L}(\boldsymbol{x}, d_{\boldsymbol{\theta}}(e_{\boldsymbol{\phi}}(\boldsymbol{x})))],$$

where $\boldsymbol{\phi}$ and $\boldsymbol{\theta}$ are the weights of the encoder and decoder networks, respectively; $e_{\boldsymbol{\phi}} : \boldsymbol{x} \mapsto \boldsymbol{z}$ and $d_{\boldsymbol{\theta}} : \boldsymbol{z} \mapsto \boldsymbol{x}$, where $\boldsymbol{z}$ is the latent representation of $\boldsymbol{x}$ and $|\boldsymbol{z}| \leq |\boldsymbol{x}|$.

In variational AEs, the input $\boldsymbol{x}$ is mapped into a probability distribution over the latent space. $e(\boldsymbol{z}|\boldsymbol{x})$ defines a distribution over the latent space that depends on the input $\boldsymbol{x}$ drawn from $p^*(\mathbf{x})$. Altogether, $p^*(\mathbf{x})$ and $e(\boldsymbol{z}|\boldsymbol{x})$ define the joint distribution $p_e(\boldsymbol{x},\boldsymbol{z}) = e(\boldsymbol{z}|\boldsymbol{x})p^*(\mathbf{x})$, whose marginal and conditional distributions are defined as:

$$p_e(\boldsymbol{z}) = \int p_e(\boldsymbol{x},\boldsymbol{z})d\boldsymbol{x} = \int p_e(\boldsymbol{z}|\boldsymbol{x})p^*(\boldsymbol{x})d\boldsymbol{x}$$
$$p_e(\boldsymbol{x}|\boldsymbol{z}) = \frac{p_e(\boldsymbol{x},\boldsymbol{z})}{p_e(\boldsymbol{z})}.$$

Since the representation $\boldsymbol{z}$ of $\boldsymbol{x}$ should maintain as much as possible the "amount of information" held in $\boldsymbol{x}$, the mutual information $I(\boldsymbol{x};\boldsymbol{z})$ can be used to measure the representation $\boldsymbol{z}$ of $\boldsymbol{x}$. Specifically, for any distribution $q(\boldsymbol{z})$ in the latent space, the mutual information between $p_e(\boldsymbol{z})$ and $p^*(\boldsymbol{x})$ can be bounded below as:

$$I(\boldsymbol{x};\boldsymbol{z}) = \mathrm{KL}(p_e(\boldsymbol{x},\boldsymbol{z})||p^*(\boldsymbol{x})p_e(\boldsymbol{z})) \leq \mathbb{E}[\mathrm{KL}(e(\boldsymbol{z}|\boldsymbol{x})||q(\boldsymbol{z}))],$$

where $\mathrm{KL}(\cdot)$ is the Kullback–Leibler divergence [2] between two distributions. $I(\boldsymbol{x};\boldsymbol{z})$ can be also bounded above, for any conditional distribution $d(\boldsymbol{x}|\boldsymbol{z})$, as:

$$I(\boldsymbol{x};\boldsymbol{z}) = \mathrm{KL}(p_e(\boldsymbol{x},\boldsymbol{z})||p^*(\boldsymbol{x})p_e(\boldsymbol{z})) \geq \mathbb{E}\left[\log\left(\frac{d(\boldsymbol{x}|\boldsymbol{z})}{p^*(\boldsymbol{x})}\right)\right].$$

Combining the provided definitions, we obtain that:

$$\mathbb{E}\left[\log\left(\frac{d(\boldsymbol{x}|\boldsymbol{z})}{p^*(\boldsymbol{x})}\right)\right] \leq I(\boldsymbol{x};\boldsymbol{z}) \leq \mathbb{E}[\mathrm{KL}(e(\boldsymbol{z}|\boldsymbol{x})||q(\boldsymbol{z}))].$$

The lower bound can be further decomposed by means of algebraic manipulations as

$$\mathbb{E}\left[\log\left(\frac{d(\boldsymbol{x}|\boldsymbol{z})}{p^*(\boldsymbol{x})}\right)\right] = \mathbb{E}[\log(d(\boldsymbol{x}|\boldsymbol{z})) + H(p^*(\boldsymbol{x}))],$$

where $H(p^*(\boldsymbol{x}))]$ is the entropy of $p^*(\boldsymbol{x})$. By following the definition provided in [1], the ELBO term, which is the measure maximised during the training of VAE, can be written as:

$$\mathrm{ELBO} = -\mathrm{KL}(p_e(\mathbf{z})||q(\mathbf{z})) - H(p^*(\boldsymbol{x})) - \mathbb{E}[\mathrm{KL}(p_e(\boldsymbol{x}|\boldsymbol{z})||d(\boldsymbol{x}|\boldsymbol{z}))].$$

In MMDVAE [1], $\mathrm{KL}(p_e(\mathbf{z})||q(\mathbf{z}))$ is multiplied by a positive factor λ and $I(\boldsymbol{x};\boldsymbol{z})$, weighted by a positive factor α, is added to the ELBO term, obtaining:

$$\begin{aligned}\mathrm{ELBO} = &-\lambda\mathrm{KL}(p_e(\boldsymbol{z})||q(\boldsymbol{z})) \\ &- H(p^*(\boldsymbol{x}) \\ &- \mathbb{E}[\mathrm{KL}(p_e(\boldsymbol{x}|\boldsymbol{z})||d(\boldsymbol{x}|\boldsymbol{z}))] \\ &+ \alpha I(\boldsymbol{x};\boldsymbol{z}).\end{aligned}$$

By applying algebraic manipulations, the ELBO term of MMDVAE can be written as:

$$\begin{aligned}\mathrm{ELBO} = &\,\mathbb{E}[\log(d(\boldsymbol{x}|\boldsymbol{z}))] \\ &- (\alpha+\lambda-1)\mathrm{KL}(p_e(\boldsymbol{z})||q(\boldsymbol{z})) \\ &- (1-\alpha)\mathbb{E}[\mathrm{KL}(p_e(\boldsymbol{z}|\boldsymbol{x})||q(\boldsymbol{z})).\end{aligned} \tag{1}$$

In MMDVAE, the term $\mathrm{KL}(p_e(\boldsymbol{z})||q(\boldsymbol{z}))$ is replaced with $\mathrm{DSD}(p_e(\boldsymbol{z})||q(\boldsymbol{z}))$, where $DSD(\cdot)$ is a general strict divergence function. $\mathrm{DSD}(p_e(\boldsymbol{z})||q(\boldsymbol{z})) = 0$ if and only if $p_e(\cdot) = q(\cdot)$. Notice that, the KL is a strict divergence function. MMDVAE exploits the Maximum Mean Discrepancy $\mathrm{MMD}(\cdot)$ divergence function [3]. A kernel trick is used to define the following divergence function between two distributions $p_e(\boldsymbol{z})$ and $q(\boldsymbol{z})$:

$$\begin{aligned}\mathrm{MMD}(p_e(\boldsymbol{z})||q(\boldsymbol{z})) = &\,\mathbb{E}_{p_e(\boldsymbol{z}),p(\boldsymbol{z'})}[\mathcal{K}(\boldsymbol{z},\boldsymbol{z'})] \\ &+ \mathbb{E}_{q(\boldsymbol{z}),q(\boldsymbol{z'})}[\mathcal{K}(\boldsymbol{z},\boldsymbol{z'})] \\ &- 2\mathbb{E}_{p_e(\boldsymbol{z}),q(\boldsymbol{z'})}[\mathcal{K}(\boldsymbol{z},\boldsymbol{z'})],\end{aligned}$$

where $\mathcal{K}(\boldsymbol{z},\boldsymbol{z'})$ can be any desired universal kernel. Here, we considered the Gaussian kernel

$$\mathcal{K}(\boldsymbol{z},\boldsymbol{z'}) = e^{-\frac{||\boldsymbol{z}-\boldsymbol{z'}||}{2\sigma^2}}.$$

We extended the ELBO term shown in Eq. 1 such that multiple Gaussian distributions can be used in the latent representation $\boldsymbol{z}$. In addition, we introduced a learnable mixture distribution for $q(\boldsymbol{z})$, whereas $p_e(\boldsymbol{z}|\boldsymbol{x})$ is defined to be a learnable mixture distribution with the same number of components.

In GMVAE [4], the encoder function outputs the following two conditional distributions $e(\boldsymbol{z}, y|\boldsymbol{x})$ and $e(\boldsymbol{z}|\boldsymbol{x}, y)$, where $y \in \{1, \ldots, K\}$ is a categorical random variable and K corresponds to the number of desired Gaussian distributions. We obtain that

$$\begin{aligned} p_e(\boldsymbol{z}, y|\boldsymbol{x}) &= \frac{p_e(\boldsymbol{z}, y, \boldsymbol{x})}{p^*(\boldsymbol{x})} \\ &= e(\boldsymbol{z}|y, \boldsymbol{x})e(y|\boldsymbol{x}). \end{aligned}$$

Since $p_e(\boldsymbol{z}, y|\boldsymbol{x})$ is fully determined by the output distributions of the encoder, we can refer to $p_e(\boldsymbol{z}, y|\boldsymbol{x})$ with $e(\boldsymbol{z}, y|\boldsymbol{x})$.

Modelling $e(y|\boldsymbol{x})$ as a categorical distribution that can assume values in $\{1, \ldots, K\}$, and $e(\boldsymbol{z}|\boldsymbol{x}, y)$ as a diagonal Gaussian distribution for each possible value assumed by y, the marginal conditional distribution $p_e(\boldsymbol{z}|\boldsymbol{x})$ is a Gaussian mixture distribution of K components, namely:

$$p_e(\boldsymbol{z}|\boldsymbol{x}) = \sum_{y=1}^{K} e(\boldsymbol{z}|y, \boldsymbol{x})e(y|\boldsymbol{x}).$$

Similarly, $q(\boldsymbol{z})$ is modelled as a Gaussian mixture distribution by using another variable $y \in \{1, \ldots, K\}$, with a categorical distribution $q(y)$, and considering the conditional distribution $q(\boldsymbol{z}|y)$ as a diagonal Gaussian distribution for each possible value of y. The ELBO term of GMVAE is:

$$ELBO = \mathbb{E}\left[\mathbb{E}\left[d(\boldsymbol{x}|y, \boldsymbol{z}) - \log\left(\frac{e(\boldsymbol{z}, y|\boldsymbol{x})}{q(\boldsymbol{z}, y)}\right)\right]\right],$$

and it can be rewritten by using the notation proposed in [1] and by algebraic manipulations as:

$$\begin{aligned} ELBO &= -\mathrm{KL}(p_e(\boldsymbol{z}, y)||q(\boldsymbol{z}, y)) \\ &= -\mathbb{E}[\mathrm{KL}(p_e(\boldsymbol{x}|\boldsymbol{z}, y)||d(\boldsymbol{x}|\boldsymbol{z}, y))] \\ &= -H(p^*(\boldsymbol{x})). \end{aligned}$$

Starting from this definition, we can add the mutual information $I(\boldsymbol{x}; (y, \boldsymbol{z}))$ term, weighted by a positive scalar factor α, and $\mathrm{KL}(p_e(\boldsymbol{z}, y)||q(\boldsymbol{z}, y))$ is weighted by a positive factor λ, obtaining:

$$\begin{aligned} \mathrm{ELBO} = &-\lambda \mathrm{KL}(p_e(\boldsymbol{z}, y)||q(\boldsymbol{z}, y)) \\ &- H(p^*(\boldsymbol{x})) \\ &- \mathbb{E}[\mathrm{KL}(p_e(\boldsymbol{x}|\boldsymbol{z}, y)||d(\boldsymbol{x}|\boldsymbol{z}, y))] \\ &+ \alpha I(\boldsymbol{x}; (y, \boldsymbol{z})), \end{aligned}$$

where

$$I(\boldsymbol{x}; (y, \boldsymbol{z})) = \mathbb{E}\left[\log \frac{p_e(\boldsymbol{x}, y, \boldsymbol{z})}{p^*(\boldsymbol{x})p_e(\boldsymbol{z}, y))}\right].$$

By applying algebraic manipulations, we can rewrite the ELBO term as:

$$\begin{aligned} \mathrm{ELBO} = &\mathbb{E}[\log(d(\boldsymbol{x}|\boldsymbol{z}, y))] \\ &- (\alpha + \lambda - 1)\mathrm{KL}(p_e(\boldsymbol{z}, y)||q(\boldsymbol{z}, y)) \\ &- (1 - \alpha)\mathbb{E}[\mathrm{KL}(p_e(\boldsymbol{z}, y|\boldsymbol{x})||q(\boldsymbol{z}, y))]. \end{aligned}$$

$\mathrm{KL}(p_e(\boldsymbol{z}, y)||q(\boldsymbol{z}, y))$ can be further decomposed as:

$$\mathrm{KL}(p_e(\boldsymbol{z}, y)||q(\boldsymbol{z}, y)) = \mathbb{E}[\mathrm{KL}(p_e(y|\boldsymbol{z})||q(y|\boldsymbol{z}))] + \mathrm{KL}(p_e(\boldsymbol{z})||q(\boldsymbol{z})),$$

so that the ELBO can be written as:

$$\begin{aligned}\mathrm{ELBO} &= \mathbb{E}[\log(d(\boldsymbol{x}|\boldsymbol{z}, y))] \\ &- (\alpha + \lambda - 1)\mathrm{KL}(p_e(\boldsymbol{z})||q(\boldsymbol{z})) \\ &- (1 - \alpha)\mathbb{E}[\mathrm{KL}(p_e(\boldsymbol{z}, y|\boldsymbol{x})||q(\boldsymbol{z}, y))].\end{aligned}$$

As in MMDVAE (see Eq. 1), we can replace $\mathrm{KL}(p_e(\boldsymbol{z})||q(\boldsymbol{z}))$ with a general strict divergence function. We considered the $\mathrm{MMD}(\cdot)$ term, obtaining the a general formulation for all the five AEs:

$$\begin{aligned}\mathrm{ELBO} &= \mathbb{E}[\log(d(\boldsymbol{x}|\boldsymbol{z}, y))] \\ &- (\alpha + \lambda - 1)\mathrm{MMD}(p_e(\boldsymbol{z})||q(\boldsymbol{z})) \\ &- (1 - \alpha)\mathbb{E}[\mathrm{KL}(p_e(\boldsymbol{z}, y|\boldsymbol{x})||q(\boldsymbol{z}, y))].\end{aligned} \quad (2)$$

We modified the $\mathrm{MMD}(p_e(\boldsymbol{z})||q(\boldsymbol{z}))$ such that it is not necessary to sample from the Gaussian mixture distribution $e(\boldsymbol{z}|\boldsymbol{x})$ or from the posterior $q(\boldsymbol{z})$. Our modification allows for sampling from the single Gaussian distributions that form the mixtures. We used the the reparametrization trick proposed in [5] so that $\mathrm{MMD}(p_e(\boldsymbol{z})||q(\boldsymbol{z}))$ can be approximated. Specifically, $\mathbb{E}_{p_e(\boldsymbol{z}), p(\boldsymbol{z'})}[\mathcal{K}(\boldsymbol{z}, \boldsymbol{z'})]$ can be approximated as:

$$\frac{1}{N^2}\sum_{i=1}^{N}\sum_{j=1}^{N}\sum_{y=1}^{K}\sum_{y'=1}^{K} p_e(y, x_i)p_e(y', x'_j)\mathbb{E}_{p_e(\boldsymbol{z}|x_i, y), p_e(\boldsymbol{z'}|x'_j, y')}[\mathcal{K}(\boldsymbol{z}, \boldsymbol{z'})],$$

where N is the number of provided samples (i.e., cells). The approximation of $\mathbb{E}_{q(\boldsymbol{z}), q(\boldsymbol{z'})}[\mathcal{K}(\boldsymbol{z}, \boldsymbol{z'})]$ is:

$$\sum_{y=1}^{K}\sum_{y'=1}^{K} q(y)q(y')\mathbb{E}_{q(\boldsymbol{z}|y), q(\boldsymbol{z'}|y')}[\mathcal{K}(\boldsymbol{z}, \boldsymbol{z'})].$$

Finally, $\mathbb{E}_{p_e(\boldsymbol{z}), q(\boldsymbol{z'})}[\mathcal{K}(\boldsymbol{z}, \boldsymbol{z'})]$ is approximated as:

$$\sum_{i=1}^{N}\sum_{y=1}^{K}\sum_{y'=1}^{K} p_e(y|x_i)q(y')\mathbb{E}_{p_e(\boldsymbol{z}|y, x_i), q(\boldsymbol{z'}|y')}[\mathcal{K}(\boldsymbol{z}, \boldsymbol{z'})].$$

To calculate the ELBO function described in Eq. 2, $\mathbb{E}[\mathrm{KL}(p_e(\boldsymbol{z}, y|\boldsymbol{x})||q(\boldsymbol{z}, y))]$ must be computable. We can rewrite it as follows:

$$\begin{aligned}\mathbb{E}[\mathrm{KL}(p_e(\boldsymbol{z}, y|\boldsymbol{x})||q(\boldsymbol{z}, y))] &= \mathbb{E}\left[\log\frac{p_e(\boldsymbol{z}|y, \boldsymbol{x})p_e(y|\boldsymbol{x})}{q(\boldsymbol{z}|y)q(y)}\right] \\ &= \mathbb{E}\left[\log\frac{p_e(\boldsymbol{z}|y, \boldsymbol{x})}{q(\boldsymbol{z}|y)}\right] + \mathbb{E}\left[\mathbb{E}\left[\log\frac{p_e(y|\boldsymbol{x})}{q(y)}\right]\right] \\ &= \mathbb{E}[\mathrm{KL}(p_e(y|\boldsymbol{x})||q(y))] + \mathbb{E}[\mathbb{E}[\mathrm{KL}(p_e(\boldsymbol{z}|\boldsymbol{x}, y)||q(\boldsymbol{z}|y))]].\end{aligned}$$

Considering the weights of $q(\boldsymbol{z})$ fixed to a uniform distribution, $\mathbb{E}[\mathrm{KL}(p_e(y|\boldsymbol{x})||q(y))]$ can be written as:

$$\mathbb{E}[\mathrm{KL}(p_e(y|\boldsymbol{x})||q(y))] = \mathbb{E}\left[\sum_{y=1}^{K} p_e(y|\boldsymbol{x}) \log(p_e(y|\boldsymbol{x}))\right] + \log(K).$$

On the contrary, when the weights are learnable, $\mathbb{E}[\mathrm{KL}(p_e(y|\boldsymbol{x})||q(y))]$ can be analytically calculated as:

$$\mathbb{E}[\mathrm{KL}(p_e(y|\boldsymbol{x})||q(y))] = \mathbb{E}\left[\sum_{y=1}^{K} p_e(y|\boldsymbol{x}) \log(p_e(y|\boldsymbol{x})) - \sum_{y=1}^{K} p_e(y|\boldsymbol{x}) \log(q(y))\right].$$

Finally, $\mathrm{KL}(p_e(\boldsymbol{z}|\boldsymbol{x}, y)||q(\boldsymbol{z}|y))$ can be calculated by the following approximation:

$$\mathrm{KL}(p_e(\boldsymbol{z}|\boldsymbol{x}, y)||q(\boldsymbol{z}|y)) = \mathbb{E}\left[\log \frac{p_e(\boldsymbol{z}|\boldsymbol{x}, y)}{q(\boldsymbol{z}|y)}\right] \approx \log \frac{p_e(\boldsymbol{z}|\boldsymbol{x}, y)}{q(\boldsymbol{z}|y)}$$

Additional Files

Additional file 2 — Excel file of the metrics calculated for the PBMC datasets

Each tab is related to a tested approach and shows the calculated metrics and used method.

Additional file 3 — Excel file of the metrics calculated for the PIC datasets

Each tab is related to a tested approach and shows the calculated metrics and used method.

Additional file 4 — Excel file of the metrics calculated for the MCA datasets

Each tab is related to a tested approach and shows the calculated metrics and used method.

References

[1] Zhao, S., Song, J., Ermon, S.: Infovae: Balancing learning and inference in variational autoencoders. In: Proceedings of the AAAI Conference on Artificial Intelligence, vol. 33, pp. 5885–5892 (2019)

[2] Kullback, S., Leibler, R.A.: On information and sufficiency. Ann Math Statist. **22**(1), 79–86 (1951). doi:10.1214/aoms/1177729694

[3] Gretton, A., Borgwardt, K., Rasch, M., Schölkopf, B., Smola, A.J.: A kernel method for the two-sample-problem. In: Proceedings of the Conference on Advances in Neural Information Processing Systems, pp. 513–520 (2007)

[4] Grønbech, C.H., Vording, M.F., Timshel, P.N., Sønderby, C.K., Pers, T.H., Winther, O.: scVAE: Variational auto-encoders for single-cell gene expression data. Bioinformatics (2020). doi:10.1093/bioinformatics/btaa293

[5] Kingma, D.P., Welling, M.: Auto-encoding variational bayes. arXiv preprint arXiv:1312.6114 (2013)
